# Adolescents do not benefit from universal school-based mindfulness interventions: a reanalysis of Dunning et al. (2022)

**DOI:** 10.3389/fpsyg.2024.1384531

**Published:** 2024-06-13

**Authors:** Brian Galla, Aishwarya Karanam, Avital Pelakh, Simon B. Goldberg

**Affiliations:** ^1^Department of Health and Human Development, University of Pittsburgh, Pittsburgh, PA, United States; ^2^Learning Research and Development Center, University of Pittsburgh, Pittsburgh, PA, United States; ^3^Department of Psychology, University of Pittsburgh, Pittsburgh, PA, United States; ^4^Department of Counseling Psychology, University of Wisconsin-Madison, Madison, WI, United States; ^5^Center for Healthy Minds, University of Wisconsin-Madison, Madison, WI, United States

**Keywords:** universal intervention, mindfulness, adolescent, prevention, meta-analysis

## Abstract

Are universal school-based mindfulness interventions an effective way to reduce risk for mental disorders and improve adolescents' lives? To answer this question, we reanalyzed data from Dunning et al.'s (2022) meta-analysis of randomized controlled trials of mindfulness interventions delivered to children and adolescents. Though Dunning et al. (2022) reported some benefits of universal mindfulness interventions, their analysis did not examine adolescents separately from children. Consequently, their conclusions may not entirely reflect the effectiveness of universal mindfulness interventions specifically for adolescents, a developmental period when mental disorders are known to increase. Using their open-access data tables, we tested impacts of 22 randomized controlled trials (*N* = 16,558) on eight outcome categories—anxiety/stress, attention, depression, executive functioning, mindfulness, negative behavior, social behavior, and wellbeing—at immediate post-test and longest follow-up. Our reanalysis shows that when compared to passive controls, mindfulness interventions significantly reduced trait mindfulness (*d* = −0.10). When compared to active controls, mindfulness interventions significantly improved anxiety/stress (*d* = 0.17) and wellbeing (*d* = 0.10). When compared to all controls combined, mindfulness interventions did not significantly improve any outcome (*d*s = 0.01 to 0.26). No effects of mindfulness interventions were observed at follow-up assessment. Overall, results of our analysis cast doubt about the value of existing school-based mindfulness interventions as a universal prevention strategy for adolescents.

## 1 Introduction

Mindfulness training is sweeping across the schooling system (Roeser et al., 2023). Tens of thousands of adolescents now pay attention to their breath, rein in wandering thoughts, and non-judgmentally observe emotional states as part of their schools' curricula. The aim is to build skills and attitudes that can prevent mental health problems (Davidson et al., 2012; Kuyken et al., 2017). For maximum impact, mindfulness training is often delivered universally to all adolescents, regardless of individual risk (Roeser et al., 2020).

The high societal burden of mental health disorders demands effective prevention strategies. Adolescence presents a strategic time to intervene. During this period, the risk for depression and other mental disorders increases (Merikangas et al., 2010). Moreover, research indicates that many adults with depression first experience symptoms as teenagers (Pine et al., 1999; Kim-Cohen et al., 2003). This suggests that intervening earlier in life has the potential to prevent years of suffering and save billions lost to healthcare costs (Greenberg et al., 2021).

Universal interventions are a promising approach for achieving widespread prevention. By reaching entire populations, they act as a first line of defense against later problems. They can also be easier to deliver, cheaper, and less stigmatizing than interventions that target vulnerable individuals or groups (Dodge, 2020).

But are universal school-based mindfulness interventions (uSBMI) an effective way to prevent mental disorders and improve adolescents' lives? Recent meta-analyses paint a complicated picture. A meta-analysis by Phillips and Mychailyszyn (2022) found that uSBMIs significantly reduced anxiety (*k* = 13, *g* = 0.20, *p* = 0.012) but not depression (*k* = 15, *g* = 0.07, *p* = 0.143). However, their analysis included studies that did not use randomized designs and did not separate adolescent samples from child samples. Another meta-analysis of only randomized controlled trials (RCTs) found that school-based mindfulness interventions significantly reduced adolescents' stress (*k* = 7, *g* = 0.55, *p* < 0.001) but not anxiety (*k* = 4, *g* = 0.19, *p* = 0.25) or depression (*k* = 6, *g* = 0.20, *p* = 0.11; Fulambarkar et al., 2023). Additional analyses revealed that mindfulness was more effective when compared against passive controls (*k* = 5, *g* = 0.38, *p* < 0.05) than active controls (*k* = 5, *g* = 0.27, *p* = 0.08). But one limitation of Fulambarkar et al.'s (2023) meta-analysis is that they combined interventions that were targeted to high-risk samples with interventions that were universally administered to everyone.

A third meta-analysis by Dunning et al. (2022)—and the basis for the current reanalysis—is the most comprehensive to date. It is also the only meta-analysis so far to include results from the My Resilience in Adolescence (MYRIAD) Project, the largest-ever RCT of a uSBMI that involved over 8,000 early adolescents (Kuyken et al., 2022). Based on their analysis, Dunning et al. concluded that uSBMIs across all age groups significantly improve attention, executive function, and social behavior, and reduce negative behaviors. However, uSBMIs did not significantly impact anxiety/stress, depression, trait mindfulness, or wellbeing.

Here, we reanalyze data from Dunning et al. (2022) for two reasons. First, their dataset only incorporated RCTs, which are the gold standard designs for establishing causality. Second, and most importantly, their analysis did not include a critical test of the effect of uSBMIs in adolescent samples. They did conduct moderation tests to assess variation in effect size estimates by age, but their overall meta-analytic estimates of universal mindfulness interventions combined children and adolescent samples together. Consequently, the conclusions reported in Dunning et al. may not entirely reflect the effectiveness of uSBMIs specifically for adolescents. This can be a problem for teachers and practitioners who want to know whether mindfulness training could help the adolescents they serve. It is a problem for theory, because school-based interventions that are effective in childhood can have lower effectiveness during adolescence (Yeager et al., 2018). It is also a problem for policymakers who want to know whether resources devoted to mindfulness training are cost effective compared to other initiatives.

Thus, in the current study, we reanalyze data from Dunning et al. (2022) to provide estimates of the meta-analytic effect sizes of universally administered, school-based mindfulness interventions in adolescent samples. We examine impacts on eight outcome categories, as originally reported in Dunning et al. (2022)—anxiety and stress, attention, depression, executive functioning, trait mindfulness, negative behavior, social behavior, and wellbeing—at immediate post-test and longest follow-up.

## 2 Method

### 2.1 Open science practices

All data, code, and results are publicly available on the Open Science Framework (OSF): https://osf.io/vbg5x/.

### 2.2 Data and materials

We extracted data for our reanalysis using Tables S1, S2, and S3 in the open-access, online supplemental material from Dunning et al. (2022). Our analytic dataset included information from RCTs that evaluated a (1) universally administered, (2) school-based mindfulness intervention, (3) with adolescent samples (operationalized here as samples with a mean age ≥11 years). For trials that included follow-up assessments, we extracted data from the longest follow-up period.

The eight outcomes of interest, as reported in Dunning et al. (2022), were (1) anxiety/stress, (2) attention (e.g., sustained attention, attention problems, distraction), (3) depression, (4) executive function (e.g., response inhibition, cognitive flexibility, working memory, sustained attention, and attention problems), (5) trait mindfulness, (6) negative behavior (e.g., externalizing behaviors, aggression, hyperactivity, anger, hostility, conduct problems), (7) social behavior (e.g., shyness and prosocial behavior), and (8) wellbeing (e.g., physical wellbeing, emotional wellbeing). The outcome category called “attention” is based on a subset of executive functioning measures that focused specifically on sustained attention and distraction. All measures labeled “attention” were also included in the executive function analysis.

### 2.3 Analysis plan

We fit a series of random effects models using the R “metafor” package (Viechtbauer, 2010) to test the impact of mindfulness on each of the eight outcome categories at posttest and follow-up. We first compared uSBMIs against passive control conditions and active control conditions separately, and then against all controls combined. Meta-analytic estimates whose 95% confidence interval did not include a value of 0 were considered statistically significant and heterogeneity estimates (*Q*) with *p*-values < 0.05 were considered statistically significant.

Our analysis involved one main deviation from Dunning et al. (2022). Some of the individual RCTs included in the Dunning et al. meta-analysis used more than one measure to assess a given outcome category. In these instances, only the measure that best captured the category (as determined by a classification system created by the authors) was included in their analysis. They indicated such choices through bolded text in supplemental data Tables S2 and S3. In our main analysis, we ignored the bolding and used all available data for all outcome categories. For trials that included multiple measures of a given category, we used the “agg” function in the “MAd” package (Del Re and Hoyt, 2014) to first aggregate effect sizes within categories. As a robustness check, we reanalyzed the data using the classification strategy described by Dunning et al. (2022).

## 3 Results

### 3.1 Descriptive statistics

The reanalysis included 22 unique studies reported in 20 publications. The sample size across all 22 studies was 16,558.

On average, participants were 14.20 years old (*SD* = 1.49 years), with a minimum mean age of 11.8 years and a maximum mean age of 17.3 years.

Of the 22 unique studies, seven used passive controls, 11 used active controls, and four used both passive and active controls. The average sample size was 352.09 (*SD* = 832.87) for the mindfulness conditions, 400.55 (*SD* = 933.04) for the control conditions, and 752.64 (*SD* = 1,764.43) for the overall sample size per study. However, the median sample sizes for the mindfulness, control, and combined samples were 56, 102, and 165, respectively, which shows that the sample size means were skewed by a few larger trials (e.g., Lassander et al., 2021; Kuyken et al., 2022).

Twelve of the 22 studies included follow-up assessment. The average (longest) follow-up period was 6.92 months (*SD* = 8.03 months), with a minimum follow-up period of 3 months and a maximum follow-up period of 31 months.

### 3.2 Impact of mindfulness at posttest

Results are provided in Table 1. All forest plots (for posttest, follow-up, and robustness analyses) are available at the OSF link above.

**Table 1:** Effects of universal school-based mindfulness interventions for adolescents at post-test.

**Outcome**	**Random effects model**								**Heterogeneity**		
	***k***	***n***	**Estimate**	**SE**	***z***	***p***	**LCI**	**UCI**	***Q***	***p***	*I* ^2^
**Anxiety/stress**											
Passive controls	7	9,193	−0.01	0.04	−0.24	0.808	−0.087	0.068	6.98	0.322	21.46%
Active controls	8	1,243	0.17	0.05	3.16	0.002	0.065	0.277	3.95	0.786	4.60%
All controls	14	10,382	0.06	0.04	1.34	0.182	−0.028	0.148	19.51	0.108	48.29%
**Attention**											
Passive controls	1	115	0.57	0.19	2.99	0.003	0.196	0.944	0.00	1.000	0.00%
Active controls	4	877	0.03	0.06	0.43	0.666	−0.092	0.144	1.77	0.622	1.13%
All controls	5	992	0.14	0.11	1.27	0.206	−0.077	0.360	9.21	0.056	59.83%
**Depression**											
Passive controls	7	12,082	−0.01	0.02	−0.54	0.591	−0.048	0.027	6.57	0.362	0.04%
Active controls	5	5,475	0.07	0.04	1.64	0.100	−0.013	0.152	6.24	0.182	44.63%
All controls	10	15,177	0.01	0.02	0.69	0.487	−0.024	0.051	15.94	0.068	9.66%
**Executive function**											
Passive controls	5	8,424	0.51	0.29	1.74	0.083	−0.066	1.088	48.87	< 0.001	97.02%
Active controls	6	1,113	0.09	0.11	0.77	0.439	−0.135	0.310	13.72	0.018	72.31%
All controls	10	9,483	0.26	0.16	1.64	0.101	−0.050	0.562	57.26	< 0.001	95.75%
**Mindfulness**											
Passive controls	4	8,910	−0.10	0.02	−4.97	< 0.001	−0.144	−0.063	2.14	0.545	0.00%
Active controls	8	1,266	0.11	0.10	1.04	0.298	−0.096	0.312	20.27	0.005	61.67%
All controls	12	10,176	0.02	0.07	0.23	0.819	−0.114	0.145	33.81	< 0.001	75.24%
**Negative behavior**											
Passive controls	1	7,912	−0.02	0.02	−0.84	0.399	−0.050	0.020	0.00	1.000	0.00%
Active controls	5	645	0.06	0.09	0.70	0.487	−0.112	0.235	5.53	0.237	31.20%
All controls	6	8,557	0.03	0.06	0.52	0.600	−0.089	0.155	8.62	0.125	43.56%
**Social behavior**											
Passive controls	2	8,116	0.04	0.06	0.65	0.515	−0.080	0.160	1.52	0.218	34.26%
Active controls	4	1,018	0.05	0.06	0.78	0.435	−0.073	0.170	2.42	0.490	2.81%
All controls	5	9,013	0.02	0.02	0.85	0.393	−0.023	0.059	2.80	0.592	0.00%
**Wellbeing**											
Passive controls	4	10,236	−0.07	0.08	−0.94	0.347	−0.230	0.081	29.53	< 0.001	85.20%
Active controls	5	3,034	0.10	0.03	3.14	0.002	0.038	0.166	3.91	0.418	0.00%
All control	8	12,067	0.03	0.02	1.84	0.066	−0.002	0.067	6.18	0.519	0.00%

*k*, number of studies; *n*, number of participants; estimate, Cohen's d effect size where positive values indicate greater improvement in the mindfulness group; SE, standard error; *z, z*-statistic; *p*, significance level; LCI and UCI, lower and upper 95% confidence intervals; *Q, Q*-statistic; *I*^2^, proportion of variability explained by differences between studies.

#### 3.2.1 Passive controls

Compared to passive controls, mindfulness interventions significantly *reduced* trait mindfulness (*d* = −0.10, *p* < 0.001) but did not significantly impact anxiety/stress, depression, executive function, negative behavior, social behavior, or wellbeing (*d*s = −0.07 to 0.51, *p*s ≥ 0.083). USBMIs significantly improved attention (*d* = 0.57, *p* = 0.003), but this analysis involved only a single study with a sample of 115 participants. Heterogeneity estimates ranged from *I*^2^ = 0.00 to 97.02% and were significant for two outcomes (executive function and wellbeing).

#### 3.2.2 Active controls

Compared to active controls, mindfulness interventions significantly improved anxiety/stress (*d* = 0.17, *p* = 0.002) and wellbeing (*d* = 0.10, *p* = 0.002) but did not significantly impact attention, depression, executive function, mindfulness, negative behavior, or social behavior (*d*s = 0.03 to 0.11, *p*s ≥ 0.100). Heterogeneity estimates ranged from *I*^2^ = 0.00 to 72.31% and were significant for two outcomes (executive function and mindfulness).

#### 3.2.3 All controls

When compared to all control types combined, mindfulness did not significantly impact any of the eight outcome categories (*d*s = 0.01 to 0.26, *p*s ≥ 0.066). Heterogeneity estimates ranged from *I*^2^ = 0.00 to 95.75% and were significant for two outcomes (executive function and mindfulness).

### 3.3 Impact of mindfulness at follow-up

Mindfulness did not have a significant impact on any outcome category at follow-up compared to passive and active controls separately or in combination. Across all analyses, effect size estimates ranged from −0.18 to 0.11. See Table 2 for results.

**Table 2:** Effects of universal school-based mindfulness interventions for adolescents at follow-up.

**Outcome**	**Random effects model**								**Heterogeneity**		
	***k***	***N***	**Estimate**	**SE**	***z***	***p***	**LCI**	**UCI**	***Q***	***p***	*I* ^2^
**Anxiety/stress**											
Passive controls	3	8,680	0.04	0.06	0.68	0.497	−0.071	0.146	3.44	0.179	42.87%
Active controls	2	477	0.07	0.09	0.81	0.415	−0.105	0.255	0.83	0.363	0.00%
All controls	5	9,157	0.04	0.04	0.82	0.414	−0.050	0.121	5.01	0.287	28.90%
**Attention**											
Passive controls	0	-	-	-	-	-	-	-	-	-	-
Active controls	1	17	−0.18	0.51	−0.36	0.722	−1.178	0.816	0.00	1.000	0.00%
All controls	1	17	−0.18	0.51	−0.36	0.722	−1.178	0.816	0.00	1.000	0.00%
**Depression**											
Passive controls	5	11,737	−0.01	0.02	−0.58	0.561	−0.049	0.027	8.15	0.086	0.00%
Active controls	3	5,134	0.03	0.03	1.02	0.307	−0.026	0.083	1.03	0.597	0.00%
All controls	6	14,491	0.00	0.02	−0.09	0.929	−0.036	0.033	9.94	0.077	2.71%
**Executive function**											
Passive controls	1	7,912	0.00	0.02	−0.04	0.965	−0.045	0.043	0.00	1.000	0.00%
Active controls	2	134	−0.06	0.14	−0.46	0.644	−0.334	0.207	0.06	0.811	0.00%
All controls	3	8,046	0.00	0.02	−0.12	0.906	−0.046	0.041	0.26	0.879	0.00%
**Mindfulness**											
Passive controls	3	8,680	−0.11	0.08	−1.32	0.188	−0.274	0.054	6.52	0.039	78.03%
Active controls	5	825	0.00	0.07	−0.04	0.968	−0.141	0.136	1.22	0.876	0.00%
All controls	8	9,505	−0.07	0.04	−1.55	0.121	−0.154	0.018	9.62	0.211	32.39%
**Negative behavior**											
Passive controls	1	7,912	0.00	0.02	0.24	0.811	−0.031	0.039	0.00	1.000	0.00%
Active controls	1	164	−0.01	0.13	−0.08	0.938	−0.262	0.242	0.00	1.000	0.00%
All controls	2	8,076	0.00	0.02	0.23	0.821	−0.031	0.039	0.01	0.913	0.00%
**Social behavior**											
Passive controls	2	8,116	0.11	0.17	0.65	0.513	−0.227	0.454	7.69	0.006	87.00%
Active controls	2	677	−0.04	0.08	−0.55	0.581	−0.197	0.110	1.12	0.289	11.01%
All controls	3	8,672	−0.03	0.02	−1.50	0.134	−0.074	0.010	1.41	0.494	0.00%
**Wellbeing**											
Passive controls	4	10,236	−0.07	0.06	−1.23	0.217	−0.181	0.041	11.43	0.010	70.34%
Active controls	5	3,034	0.04	0.03	1.25	0.213	−0.023	0.104	1.32	0.858	0.00%
All controls	8	12,067	−0.01	0.02	−0.55	0.582	−0.044	0.025	1.16	0.992	0.00%

*k*, number of studies; *n*, number of participants; estimate, Cohen's *d* effect size where positive values indicate greater improvement in the mindfulness group; SE, standard error; *z, z*-statistic; *p*, significance level; LCI and UCI, lower and upper 95% confidence intervals; *Q, Q*-statistic; *I*^2^, proportion of variability explained by differences between studies.

### 3.4 Robustness tests

Results of robustness checks—using only the subset of measures derived from a coding hierarchy described in Dunning et al. (2022)—showed the same pattern of statistically significant effects at posttest and follow-up as our main analyses. Full results are available on the OSF page.

## 4 Discussion

Our reanalysis of open-access data from Dunning et al. (2022) shows that, overall, universal school-based mindfulness interventions do not reduce adolescents' anxiety and stress, depression, or negative behaviors, and likewise, do not increase attention, executive function, mindfulness, social behavior, or wellbeing at posttest or follow-up. With only three exceptions (described in a moment) these findings were consistent when comparing mindfulness interventions against passive controls, active controls, or all controls combined. Different approaches to aggregating outcomes within individual RCTs produced similar results.

Though most comparisons between mindfulness and controls were not statistically significant, three were significant. USBMIs were found to significantly reduce trait mindfulness compared to passive controls—which, according to Dunning et al., involved either “no intervention, usual practice or wait list” (p. 2). This counterintuitive result means that adolescents' levels of trait mindfulness would have been better off had they not gotten anything vs. getting a uSBMI. Conversely, uSBMIs were found to significantly improve anxiety/stress and wellbeing compared to active controls—alternative activities to mindfulness that involved “attention placebos…[or] ingredients targeting change in one or more outcomes” (Dunning et al., 2022, p. 2).

What do we make of these exceptions? Is it really the case that uSBMIs simultaneously reduce trait mindfulness when compared to nothing, improve anxiety/stress and wellbeing when compared to active controls, and have no effect on any other measured outcome? This is certainly a possibility. Perhaps these exceptions speak to a specificity of mindfulness training (e.g., it reduces anxiety but not depression) that only emerges when looking across many trials. But it is still hard to understand why trait mindfulness decreases following uSBMIs, which are, of course, designed to increase mindfulness.

We think these exceptions also need to be viewed in relationship to the totality of findings. Across 48 separate analyses (eight outcome categories × three control types × two time points), 94% were not statistically significant and only 6% were. Like all null hypothesis testing, meta-analysis is not immune to false positives. Due to the number of tests conducted, there is a chance that the small number of statistically significant effects are Type I errors.

Another possibility is that classifying controls as either passive or active is more difficult in school settings than highly controlled, lab-based clinical trials. For example, Dunning et al. (2022) labeled treatment as usual (or “usual practice” in their terms) as a passive control. This means that the very large MYRIAD Project trial (Kuyken et al., 2022) was listed as having used a passive control even though usual practice involved students receiving their schools' existing social-emotional learning programs. Given the challenges involved in classifying control types, it seems that the combined analysis—which showed no effect of mindfulness training on any outcome—may give the best overall impression of what happens when pre-teens and teens get a uSBMI.

The findings reported here are consistent with some of the conclusions originally reported in Dunning et al. (2022). For example, even though Dunning et al. (2022) combined all age groups in their analysis of universal mindfulness interventions, we both found that these interventions did not improve anxiety/stress, depression, mindfulness, or wellbeing at posttest and follow-up. But the results of our reanalysis diverge from Dunning et al. (2022) in important ways. They reported significant increases in executive function and attention from universal interventions at posttest, whereas we did not. They also reported significant improvements in negative behavior and social behavior at posttest, but we found no such effects. These discrepancies demonstrate the value of our reanalysis for answering the more targeted theory, practice, and policy relevant question of whether universal mindfulness interventions delivered in schools work specifically for adolescents.

Our findings raise serious doubts about the value of school-based mindfulness interventions as a universal prevention strategy for adolescents. The straightforward answer to the question of whether these interventions reliably reduce risk and improve adolescents' lives is no, they do not.

There are many possible reasons why existing uSBMIs have not been effective for adolescents. One consistent finding in the literature is that teens do not do the recommended meditation practices outside the classroom—activities crucial for nurturing mindfulness skills (Galla, 2024). If adolescents do not spend enough time developing mindfulness, then it will not be much help for managing stress when it matters. It is also possible that classroom teachers lack the expertise needed to administer uSBMIs successfully. One study found that after 8 weeks of personal mindfulness training and 4 days of intervention implementation training, only 29% of teachers attained the minimum competency level for delivering mindfulness to their students (Crane et al., 2020). These problems could be addressed with more rigorous teacher training and allocating more time for students to practice mindfulness within the school day. But it is worth contemplating whether resolving these issues justifies diverting resources from other programs or policies that could more reliably enhance adolescents' long-term wellbeing.

Our analysis has several limitations. The dataset from Dunning et al. (2022) is now somewhat out of date. Their literature search spanned up to January 2022, and so the dataset does not include RCTs whose results were made available during much of 2022 or 2023 (except for the MYRIAD Project). That said, our results are consistent with findings from at least two RCTs published after January 2022, neither of which found significant effects of mindfulness on adolescents' mental health compared to controls (Bazzano et al., 2022; Bogaert et al., 2023). Moreover, our reanalysis included a fairly large number of studies and participants, so we would not expect our conclusions to change dramatically with the addition of a small number of new studies. It is worth noting as a counterpoint that an RCT by Scafuto et al. (2022) did find benefits of mindfulness training on adolescents' internalizing and externalizing behaviors, and social and thought problems.

Another limitation is that only 12 of the 22 RCTs included follow-up assessments, so meta-analytic estimates on long-term effects of uSBMIs should be treated with caution. Similarly, some of the effect sizes estimates were based on single studies (e.g., uSBMIs vs. passive controls for the attention outcome at immediate posttest).

Universal school-based mindfulness interventions have been administered to adolescents for nearly 20 years. Results of our reanalysis of meta-analytic data suggest that it is time to reconsider their value as a public health prevention strategy for youth.

## Data availability statement

Publicly available datasets were analyzed in this study. This data can be found at: https://osf.io/vbg5x/.

## Ethics statement

Ethical approval was not required for the study involving humans in accordance with the local legislation and institutional requirements. Written informed consent to participate in this study was not required from the participants or the participants' legal guardians/next of kin in accordance with the national legislation and the institutional requirements.

## Author contributions

BG: Conceptualization, Data curation, Investigation, Project administration, Supervision, Writing – original draft, Writing – review & editing. AK: Data curation, Writing – review & editing. AP: Data curation, Formal analysis, Writing – review & editing. SG: Conceptualization, Data curation, Formal analysis, Writing – review & editing.
